# Function of Immune Checkpoints in IgG4–Related Disease with Lacrimal Gland Involvement: Clinical Features, Serum IgG4 Level, Immunohistochemical Landscape, and Treatment Responses

**DOI:** 10.3390/ijms26073021

**Published:** 2025-03-26

**Authors:** Dong Hyuck Bae, Yoo Ri Kim, WooKyeom Yang, Gwang Il Kim, Helen Lew, Jongman Yoo

**Affiliations:** 1 R&D Institute, Organoidsciences Ltd., Seongnam 13488, Gyeonggi-do, Republic of Korea; bdhyuck@oragnoidrx.com (D.H.B.); wk_yang@organoidrx.com (W.Y.); 2 Department of Microbiology, CHA University School of Medicine, Seongnam 13488, Gyeonggi-do, Republic of Korea; 3 CHA Organoid Research Center, CHA University, Seongnam 13488, Gyeonggi-do, Republic of Korea; 4 Department of Ophthalmology, Bundang CHA Medical Center, CHA University, Seongnam 13496, Gyeonggi-do, Republic of Korea; a206014@chamc.co.kr; 5 Gangnam Severance Hospital, Yonsei University College of Medicine, Seoul 06273, Gyeonggi-do, Republic of Korea; 6 Department of Pathology, Bundang CHA Medical Center, CHA University, Seongnam 13496, Gyeonggi-do, Republic of Korea; blacknw@cha.ac.kr

**Keywords:** IgG4–RD, lacrimal gland, multiplex–IF, CODEX, spatial analysis

## Abstract

IgG4–related disease (IgG4–RD) is an autoimmune condition marked by IgG4–positive plasma cell infiltration, causing inflammation, fibrosis, and tumor–like lesions, especially in the lacrimal gland (LG). Current diagnostic criteria, based primarily on serum IgG4 levels, face limitations in predicting clinical outcomes and treatment responses. To address this, we conducted a multiplex immaunohistochemical analysis of LG tissues to assess immune checkpoint interactions and immune cell distribution in relation to mass size, fibrosis, and treatment response. Our findings revealed that PD–L1 (Programmed Death–Ligand 1), an immune checkpoint molecule, plays a key role in shaping an immunosuppressive environment that varies by clinical group. In non–responsive patients, increased co–expression of PD–L1 and CD11c+ dendritic cells (DCs) suggested a link to treatment resistance. Spatial analysis highlighted more active immune responses in non–fibrotic areas, while fibrotic regions exhibited stabilized immune interactions driven by PD–L1 expression. These results indicate that PD–L1 contributes to immune regulation and disease progression in IgG4–RD and emphasize its potential as a therapeutic target. This study provides new insights into the immunological landscape of IgG4–RD and paves the way for the development of personalized treatment strategies.

## 1. Introduction

IgG4–related disease (IgG4–RD) is an autoimmune condition characterized by the infiltration of affected tissues by IgG4–positive plasma cells. It is a systemic disease that can affect several organs and tissues, leading to inflammation, fibrosis, and the formation of pseudotumors. IgG4–RD was initially identified in the pancreas (IgG4–related pancreatitis), but it can involve the pancreas, salivary gland, lacrimal gland, kidney, lung and retroperitoneum, giving rise to a spectrum of clinical manifestations [1].

The clinical presentation of IgG4–RD varies depending on the organs involved. Patients may experience swelling, pain, and dysfunction of affected organs. In some cases, pseudotumors or mass–like lesions develop. An elevated serum level of IgG4 is a characteristic feature, but the diagnosis is typically confirmed by radiological and histopathological examination of biopsy samples [2]. Histopathological examination of affected tissues reveals a dense lymphoplasmacytic infiltrate, fibrosis, and obliterative phlebitis (inflammation of veins). The presence of IgG4^+^ plasma cells is a hallmark of IgG4–RD [3].

Lacrimal glands (LGs) are commonly affected in autoimmune and inflammatory diseases because of their unique immunological and anatomical characteristics. The glands contain lymphoid tissue and are involved in the production of tears, which contain antimicrobial factors and immunoglobulins [4]. This immunological environment makes the lacrimal glands susceptible to immune dysregulation in autoimmune diseases such as Sjögren syndrome and Mikulicz disease (a subtype of IgG4–related disease). The susceptibility of LGs to autoimmune involvement is a result of their immunological complexity, high density of immune cells, association with systemic autoimmune diseases, presence of gland–specific antigens, and their role in maintaining ocular surface health. The immunological and anatomical characteristics of LGs make them a common target in autoimmune and inflammatory conditions affecting the eyes and surrounding tissues [4,5].

Treatment of IgG4–RD typically involves corticosteroids, such as prednisone, to suppress the inflammatory response. In some cases, immunosuppressive medications may be considered. The response to treatment can be variable, and long–term management may be required. The variable treatment responses in IgG4–RD, often reliant on systemic steroids, underscore the intricate balance between immune dysregulation and therapeutic interventions. Exploring the dynamics of treatment responses and the immunologic influence on the clinical course of IgG4–RD could enable the development of tailored treatment regimens, optimization of outcomes, and identification of novel therapeutic targets [5].

IgG4 RD, which is marked by aberrant immune responses, has been a focus of research into immune checkpoint (IC) modulation. ICs, which maintain immune homeostasis, are implicated in the pathogenesis of autoimmune diseases. Understanding these features is crucial for the development of IC inhibitors and other immunotherapies. The challenge lies in modulating the immune response to address the features of the condition, avoiding excessive immune activation in autoimmune diseases and enhancing immune recognition and response. Although our understanding of ICs in autoimmune diseases has advanced, the intricate relationship between IC and IgG4–RD, especially with LG involvement, is nascent [6].

We explored the complex relationships between LG involvement, IC dysregulation, and clinical presentation in multiple organs. Although ICs are reportedly important in several autoimmune diseases, their functions in IgG4–RD, particularly with LG involvement, are unclear. We used immunohistochemical techniques, including CODEX (CO–Detection by indEXing), to evaluate the IC landscape in LG tissues by spatial cell biology analysis. The aim was to identify IC signatures and assess their implications in IgG4–RD.

To investigate the interplay between ICs and the clinical features of IgG4–RD with LG involvement, we assessed the correlations of the serum IgG4 level, IC modulation, and ocular manifestations and evaluated the effects of ICs on treatment responses in IgG4–RD with LG involvement. The findings contribute to the development of personalized therapeutic strategies and provide insight into the pathogenesis of IgG4–RD.

## 2. Results

### 2.1. LG–Originating IgG4–RD: Characteristics, Diagnostic Methods, and Association with Clinical Manifestations

The hallmarks of IgG4–related disease in affected tissues, such as excessive B–cell proliferation and abnormal regulation of immune cells, lead to its clinical manifestations (mass, fibrosis, and proptosis), which are primarily diagnosed based on the serum level of IgG4. To explore the immunohistochemical landscape and its clinical implications, we constructed tissue microarrays (TMAs) from patients with a variety of clinical symptoms. As summarized in Table 1, the overall demographic and clinical information for each patient (including age, sex, site of involvement, serum IgG4 level, pathology findings, and treatment response) is presented. We then performed Fisher’s exact test to analyze associations between the classical diagnostic criteria for IgG4 (serum IgG4 level, IgG4/IgG ratio, and number of IgG4 positive cells) and proptosis, mass cross–sectional size, fibrosis, and treatment response (Table 2).

There were no significant associations among the clinical features according to the indices analyzed. These findings suggest the need for further studies to refine understanding and improve diagnostic and therapeutic approaches for this complex condition of IgG4–RD.

### 2.2. Validation of Immune Markers in IgG4–RD Tissues

**Figure 1:**
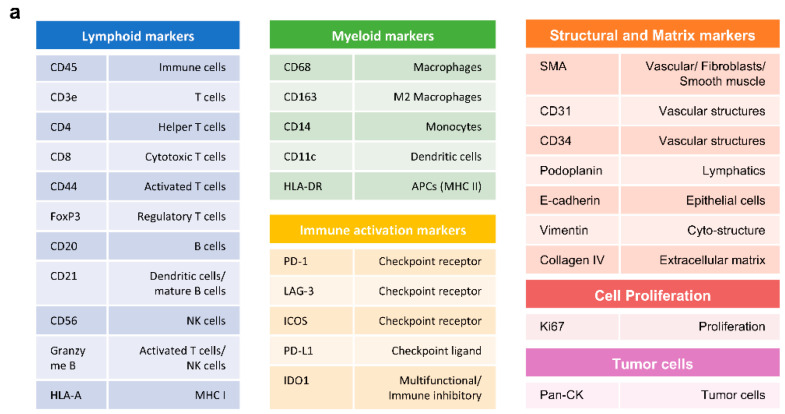

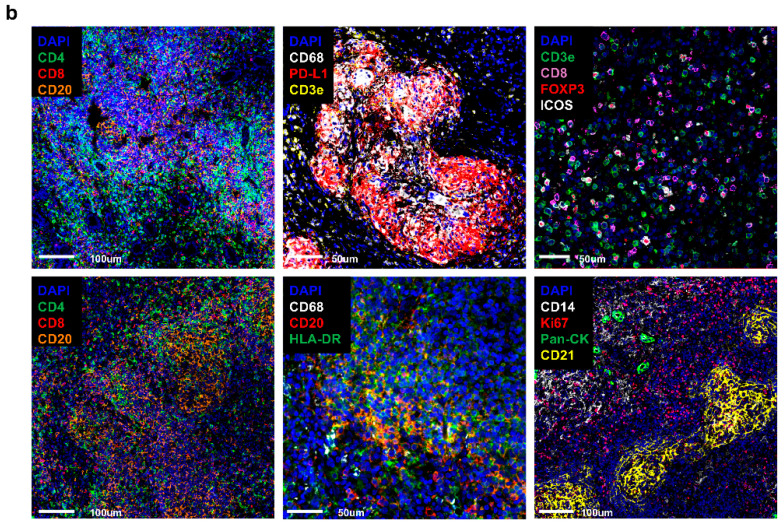
Overview and Validation of Immune Markers in IgG4–RD Tissues. (**a**) Overview of the immune marker panel used for the study, including lymphoid markers (CD45, CD4, CD8, CD20, FOXP3, CD44, Granzyme B, HLA–A), myeloid markers (CD68, CD163, CD14, CD11c, HLA–DR), immune activation markers (PD–1, LAG–3, ICOS, PD–L1, IDO1), structural markers (SMA, CD31, CD34, Podoplanin, E–cadherin, Vimentin, Collagen IV), cell proliferation markers (Ki67), and tumor cell markers (Pan–CK). (**b**) Validation results showing representative staining of immune markers in IgG4–RD tissue samples, demonstrating distinct expression patterns. The images illustrate the distribution of lymphoid markers (CD4, CD8, CD20), immune activation markers (PD–L1, ICOS), myeloid markers (CD68, CD14), and structural markers (SMA, Vimentin), indicating accurate and specific staining across various tissue types. Abbreviations: IgG4–RD, IgG4–related disease; CD, cluster of differentiation; FOXP3, forkhead box P3; PD–L1, programmed death–ligand 1; ICOS, inducible T–cell costimulator; IDO1, indoleamine 2,3–dioxygenase 1; LAG–3, lymphocyte–activation gene 3; SMA, smooth muscle actin; Pan–CK, pan–cytokeratin.

To confirm the accuracy and specificity of immune marker staining in IgG4–RD tissues, we validated 31 immune markers across different immune cell types and tissue structures. Figure 1a presents a comprehensive summary of the markers utilized, categorized into lymphoid, myeloid, immune activation, structural, and tumor cell markers. This validation demonstrated distinct localization and expression patterns that align with known tissue structures and immune cell distributions, supporting the reliability of the selected markers for further spatial analysis (Figure 1b).

### 2.3. Immune Cell Characteristics and Interactions According to Mass Size in IgG4–RD Lacrimal Gland Tissues

**Figure 2:**
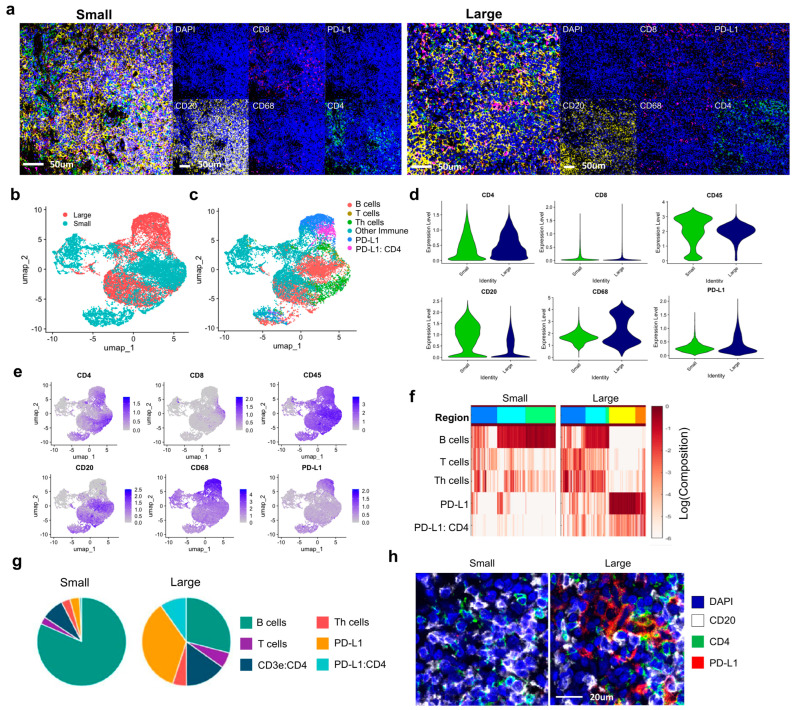
Comparison of Immune Marker Distribution Between Small and Large Mass Groups in IgG4–RD Lacrimal Gland Tissues. (**a**) Visual representation of immune cell distribution comparing small and large mass groups in IgG4–RD lacrimal gland tissues. CD20, CD4, CD8, CD68 and PD–L1 marker distributions are shown. (**b**) UMAP plot analysis of cell clusters from tissues with size of mass (**c**) group by cell immune types, (**d**,**e**) Violin plot and feature plot. (**f**) Heatmap of each group clusters, 50 μm–radius neighborhoods. (**g**) Pie chart of the relative percentage of immune cell types group by mass size groups. (**h**) Validated region images. Abbreviations: UMAP, uniform manifold approximation and projection; CD, cluster of differentiation; PD–L1, programmed death–ligand 1; Cytomap, spatial analysis toolbox; Th, T helper; PD–L1:CD4, programmed death–ligand 1 expressing CD4 cells.

Based on the criteria outlined in the Methods section (median value: 100.0 mm^2^), IgG4–RD lacrimal gland tissues were divided into small and large mass groups for analysis. Profiling of immune cell distributions between the small and large mass groups revealed distinct patterns (Figure 2a). In the small mass group, CD20+ B cells were predominantly observed, followed by CD4+ T cells. In contrast, the large mass group exhibited a distribution of immune cells, with higher proportions of CD20+ B cells, CD4+ T cells, CD8+ T cells, and CD68+ macrophages, consistent with established immune cell profiles in IgG4–RD tissues. However, further analysis focused on the expression and distribution of the immune checkpoint marker PD–L1 in CD20 and CD68–expressing areas to identify potential differences. UMAP visualization revealed heterogeneous patterns between the groups (Figure 2b,c). In particular, the large mass group showed cells expressing PD–L1 and cells co–expressing PD–L1 and CD4. The violin plots indicated an increase in CD68 expression and a slight increase in PD–L1 expression in the large group compared to the small group, although these differences were not statistically significant (Figure 2d). Feature plot analysis confirmed that PD–L1 was more widely distributed in large mass samples (Figure 2e). To further investigate how PD–L1 contributes to mass enlargement, a spatial neighborhood analysis using Cytomap was conducted with a 50 μm radius neighborhoods using raster scan option. This analysis was performed on integrated datasets, where data from all small and large lacrimal gland samples were merged within each group. The spatial neighborhood analysis revealed three distinct regions in the small mass group and five regions in the large mass group based on cellular composition. Among these, Regions 4 and 5 in the large mass group exhibited particularly high frequencies of PD–L1–expressing cells (Figure 2f). Overall, the five regions identified through spatial clustering were assigned based on the predominant immune cell types, including B cells, T cells, Th cells, PD–L1–expressing cells, and PD–L1:CD4 co–expressing cells, which reflect the key immune interactions within the tissue microenvironment. Quantitative comparison of cell type proportions showed that B cells (CD20) decreased in the large mass group, whereas T cells and Th cells increased (Figure 2g). Additionally, this image clearly demonstrates areas of co–expression between PD–L1 and CD4, providing a more accurate visualization of their spatial distribution (Figure 2h).

### 2.4. Increased Immune Interactions in Patients with Poor Treatment Response in IgG4–RD

**Figure 3:**
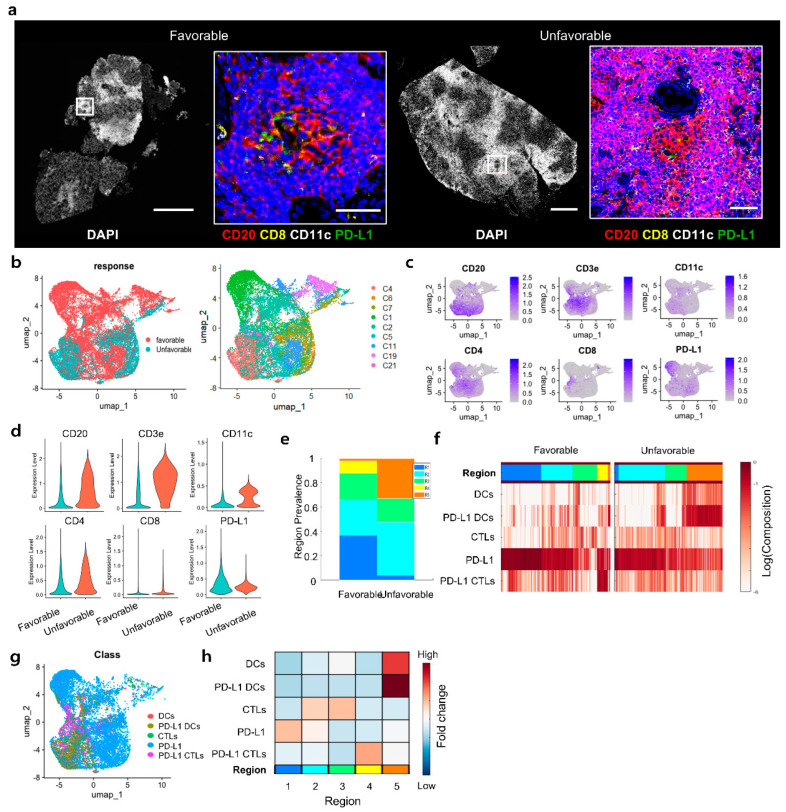
Immune Characteristics and Distribution in Patients with Poor Treatment Response. (**a**) Images show selected regions from the response group, highlighting the expression of markers CD20, CD8, CD11c, and PD–L1. (**b**) UMAP visualization categorizing patient groups based on treatment response. (**c**) Feature plot and (**d**) Violin plot showing the distribution of immune marker expression levels in both patient groups. (**e**) Spatial analysis displays the proportion of regions identified in favorable and unfavorable patients. (**f**) Heatmap from neighborhood analysis, showing the spatial interactions and distributions of immune cells. (**g**) UMAP visualization illustrates the distribution of different cell types. (**h**) Fold change analysis indicating regional differences in immune cell expression levels.

A comparison between patients who did not show improvement after 6 months of treatment (unfavorable) and those with favorable clinical outcomes revealed key differences. CD11c expression was notably increased in the unfavorable group (Figure 3a). Although the distributions of immune markers between the two groups largely overlapped (Figure 3b), the UMAP visualization was expanded with an additional plot displaying patient–specific contributions, providing a clearer understanding of individual variability. The unfavorable group showed a more localized and restricted pattern (Figure 3c). An increase in T–cell markers, such as CD3e and CD4, was observed in the unfavorable group, whereas PD–L1 expression was found to be reduced (Figure 3d). Through Cytomap analysis, five distinct spatial regions were identified based on the proximity and interactions of immune cells within the tissue (Figure 3e). Neighborhood analysis revealed that while PD–L1 was generally distributed across both groups, the frequency of CD11c+ dendritic cells (DCs) and PD–L1–expressing DCs was significantly higher in the unfavorable group (Figure 3f,g). Fold change analysis further confirmed that Region 5 had higher expression levels of DCs and PD–L1–expressing DCs in unfavorable patients (Figure 3h). However, it is important to note that the observed enrichment of PD–L1+ DCs was predominantly driven by a single patient among the three patients in the unfavorable group. This finding highlights the potential variability in the immune microenvironment across individuals. While the data provide valuable insights into the role of PD–L1+ DCs in immune regulation and treatment resistance, the small sample size limits the generalizability of these results.

### 2.5. Fibrotic and Non–Fibrotic Region Analysis in Patient Tissues

**Figure 4:**
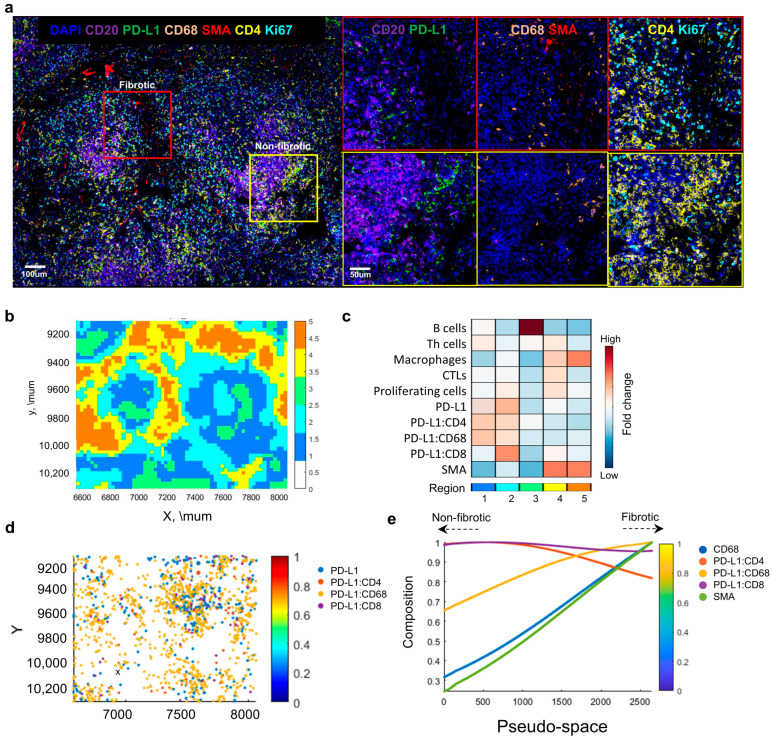
Comparative Analysis of Immune Characteristics in Fibrotic and Non–Fibrotic Regions in IgG4–RD Tissues. (**a**) Representative images showing fibrotic and non–fibrotic regions with marker expression for CD20, CD68, SMA, and PD–L1. (**b**) Positional plot of the fibrotic regions. (**c**) Fold change analysis in each region. (**d**) Positional plot of the immune cell types. (**e**) Pseudo–space plots of the fibrotic regions.

To characterize the properties of fibrotic regions in patient tissues, specific areas were selected based on marker expression (Figure 4a). Regions where SMA (smooth muscle actin) expression was observed around areas expressing CD20 and CD68 were designated as fibrotic regions, whereas regions with little or no SMA expression were classified as non–fibrotic. Spatial analysis of these selected regions revealed that the fibrotic areas mainly corresponded to Regions 4 and 5 (Figure 4b–d). Interestingly, while these areas exhibited characteristic SMA expression, immune–suppressive PD–L1 was predominantly found in Regions 1 and 2, and B cell expression was higher in Region 3. This suggests that non–fibrotic areas displayed more active immune responses compared to fibrotic regions. To further explore the transition between non–fibrotic and fibrotic regions, a pseudo–space analysis was conducted (Figure 4e). The analysis showed that as the tissue progressed toward fibrosis, the expression of SMA and CD68 increased; however, CD68 appeared to be suppressed by PD–L1. These results indicate that during the fibrotic stage, immune responses decrease, and the tissue transitions to a more stable state. Furthermore, it suggests that PD–L1 may play a role in immune responses even in the fibrotic phase.

### 2.6. Validation of Multiplex IF Analysis in Patients with IgG4–RD Developing from Nonspecific Inflammation in the LG

**Figure 5:**
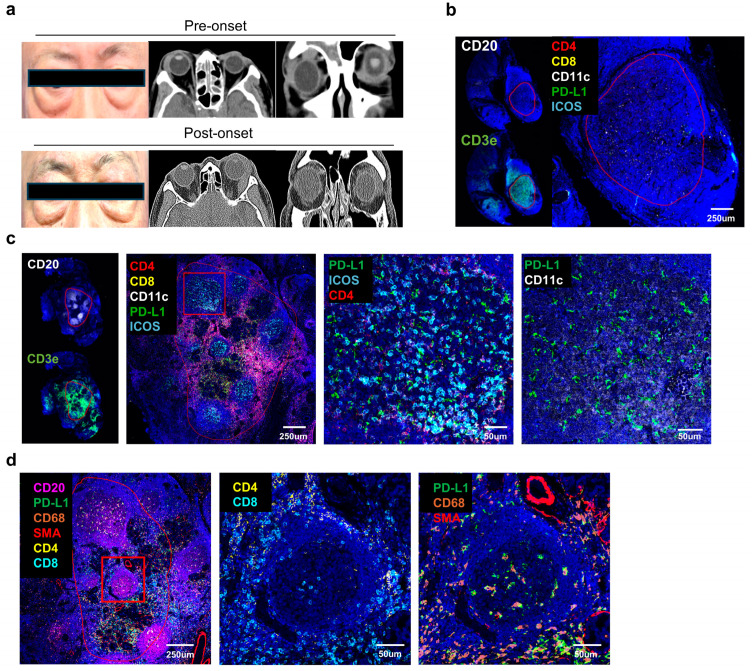
Validation in a Patient with IgG4–Related Disease Arising from Non–Specific Inflammation in the Lacrimal Gland. (**a**) Sixty–seven–year–old male patient with right eyelid swelling with proptosis (**top**). Right lacrimal gland enlargement at axial and coronary view in orbital CT (middle and right). (**Bottom**) Same patient demonstrated left eyelid swelling with proptosis six years later. Left lacrimal gland enlargement at axial and coronal view in orbital CT. (**b**) Whole image in pre–onset and (**c**) post–onset tissue. (**d**) Post-onset tissue image showing fibrotic region. Red boxes highlight fibrotic regions.

A 67–year–old male patient (#22) with right eyelid swelling with proptosis presented with right LG enlargement on axial and coronary views by orbital CT (Figure 5a). The lacrimal gland showed reactive lymphoid hyperplasia, suggestive of pseudolymphoma without fibrosis, or plasma cell proliferation. Immunohistochemical staining revealed an IgG4/IgG ratio of <0.05. The patient was subsequently treated with low–dose radiation on the right side and recovered uneventfully. However, the same patient (#23) visited the clinic for left eyelid swelling with proptosis 6 years later. Left LG enlargement was visible on axial and coronary views by orbital CT. The LG showed dense lymphoplasmacytic infiltration with mild fibrosis, suggestive of IgG4–RD. Immunohistochemical staining revealed an IgG4/IgG ratio of 0.8 with higher than 40 IgG4–positive cells per HPF. We analyzed right–side and left–side tissue samples from the same patient to validate the characteristics observed in mass size, treatment response, and fibrosis analyses of IgG4 ROD (Figure 5b–d). In the right–side tissue, CD20 expression (B cells) was minimal, and regions with CD3e expression were selected as areas of immune activity (Figure 5b). However, the overall expression of immune activation and suppression markers, such as CD4, CD8, CD11c, PD–L1, and ICOS, was not prominently observed. In contrast, left–side tissue revealed CD20 and CD3e in distinct circular patterns across multiple regions. This patient met the criteria for the large mass group, with regions showing close proximity of PD–L1 and CD4 expression, indicating active immune regions (Figure 5c). Additionally, ICOS expression was also observed, further confirming robust immune activity. The occurrence of IgG4 ROD after six years represented inflammatory disease progression in one individual, evidenced by the co–localized expression of CD11c (DCs) and PD–L1. Consistent with the findings in fibrotic areas, regions expressing SMA showed relatively lower levels of CD4 and CD8, while co–expressing cells of PD–L1 and CD68 were identified, indicating a stabilized immune response (Figure 5d). These results suggest that the PD–L1 immune checkpoint plays a significant role in various clinical manifestations of IgG4–RD in the lacrimal gland and supports the consideration of PD–L1 as an important target in IgG4–RD treatment strategies.

## 3. Discussion

IgG4–RD is an idiopathic, multi–organ inflammatory state that can manifest as chronic, relapsing, sclerosing inflammation in virtually any organ system. There is a wide range of presentations in orbital and ocular inflammation including sclerouveitis and pachymeningitis with optic neuritis resulting in permanent visual loss [7]. IgG4–RD in the orbit, in particular in the LG, typically does not show obliterative phlebitis, although the plasma cell–rich inflammatory infiltrate is often tightly perivascular, and the number of IgG4–positive plasma cells is striking [8]. Interestingly, in most patients, the serum level of IgG4 is elevated, and the orbit may be the initial or sometimes only manifestation of the disease [9].

This study includes significant findings aimed at better understanding the diagnosis and immunological characteristics of IgG4–related disease (IgG4–RD). In particular, it was confirmed that conventional diagnostic criteria (such as serum IgG4 levels and IgG4/IgG ratios) have limited correlation with clinical manifestations, emphasizing the need for new diagnostic criteria tailored to different subtypes of IgG4–RD. These results suggest that immunological patterns may vary across different patient groups, highlighting the necessity for developing more refined diagnostic approaches. Such advancements could contribute to the early detection of IgG4–RD and the formulation of accurate treatment strategies.

Autoimmune responses are characterized by the activation of autoreactive T cells and the production of autoantibodies. This process results in inflammation and tissue damage due to the immune system’s attack on healthy cells. In autoimmune diseases, IC dysregulation may contribute to excessive immune activation against self–antigens, leading to autoimmunity. ICs such as PD–1 and CTLA–4 may modulate these autoreactive responses and promote the evasion by cancer cells of immune detection and destruction [10]. The ICOS/ICOSL and PD–1/PD–L1 pathways are important in the early stages of neuromyelitis. ICOS and PD–1 have potential as therapeutic targets and biomarkers for the differential diagnosis of early–stage autoimmune neuromyelitis [10]. Inflammation and damage occur at sites of autoimmune attack, which encompass heterogeneous interactions between immune cells and stromal elements, thereby influencing disease progression. Therefore, neighbor analysis of infiltrating immune cells and the immunosuppressive microenvironment are crucial considerations in immunotherapy.

The increased expression of CD11c+ dendritic cells (DCs) and PD–L1 in non–responsive patients suggests that an immunosuppressive environment may be associated with treatment resistance (Figure 3). This highlights how interactions among these immunosuppressive cells and molecules can facilitate immune regulation and disease progression within the tissue. The fact that the interaction between PD–L1 and CD11c+ DCs is particularly pronounced in non–responsive patients suggests that they may act as key factors in immune suppression and could be considered potential targets for new therapeutic approaches.

However, we must note that our unfavorable group included only one patient, which limits the strength of these conclusions. This study is exploratory, and the observed association does not establish a direct causal link between PD–L1+ DCs and treatment resistance. Further large–scale, multi–center studies are needed to confirm our findings and to clarify whether PD–L1+ DC enrichment reflects a compensatory immunosuppressive mechanism or potentially contributes to disease progression. Our findings of increased PD–L1 expression support the notion that, beyond conventional autoimmune mechanisms, immune checkpoint pathways may play a pivotal role in the pathogenesis of IgG4–RD. Indeed, Arora et al. [11] demonstrated concurrent overexpression of PD–L1, PD–1, IDO1, and LAG3 in multiple organs (e.g., pancreas, salivary glands, lungs), and Zhang et al. [12] further suggested that PD–L1 and PD–L2 could impact Treg differentiation. These observations indicate that IgG4–RD may not be driven solely by classic autoimmunity but also by the immunomodulatory effects of checkpoint molecules. Nevertheless, current evidence remains limited by small sample sizes and a lack of large–scale prospective studies. Future research should evaluate the causal link between PD–L1 expression and key clinical features—such as disease progression, fibrosis, and therapeutic response—while also clarifying whether immune checkpoint inhibitor therapy may trigger or exacerbate IgG4–RD in susceptible individuals. Expanding the evidence base in this area is critical for refining personalized treatment approaches.

Recent evidence also indicates that immune checkpoint molecules, particularly the PD–1/PD–L1 pathway, may play a pivotal role in the pathogenesis of IgG4–related disease. Multiple studies have shown that PD–L1 is overexpressed in a range of IgG4–RD lesions, including those of the pancreas, salivary glands, and lungs, often correlating with Treg infiltration and increased fibrosis [11,12]. These findings suggest that PD–L1–mediated immune suppression contributes to local tissue remodeling and disease progression. Furthermore, recent case reports have documented new–onset or exacerbation of IgG4–RD following immune checkpoint inhibitor therapy [13,14]. Taken together, these observations imply that PD–1/PD–L1 signaling not only underpins the immunopathology of IgG4–RD but also warrants careful clinical surveillance for IgG4–RD development or flare in patients receiving anti–PD–1/PD–L1 immunotherapies. Consequently, targeting the PD–1/PD–L1 axis, with close monitoring for potential complications, may offer additional insights into personalized treatment strategies for IgG4–RD in the future.

The findings that PD–L1 plays a crucial role in immune suppression and tissue stabilization in IgG4–RD present an opportunity for new treatment strategies. The potential of PD–L1–targeted immune therapies needs to be further explored through additional studies. Understanding the differences in immune responses between fibrotic and non–fibrotic regions in particular provides important insights for developing differentiated treatment strategies. Spatial analysis using Cytomap goes beyond simple quantitative analysis of cells and offers critical insights into the interactions and spatial distribution of immune cells, helping to better understand the tissue microenvironment. This study visually confirmed that PD–L1 expression is more widely distributed in larger tumor groups, demonstrating that spatial analysis is essential for understanding disease progression and immune response patterns.

To our knowledge, there are no reports of LG–specific IgG4–RD with features similar to late–onset IgG4–RD developing from nonspecific dacryoadenitis. A middle–aged female was reported to present with swelling of the lower lid for enlargement of the right inferior rectus muscle belly [15]. She presented 6 years prior with upper eyelid swelling, and five surgical biopsies revealed inflammatory pseudotumor, chronic inflammation, inflammatory lesions, IgG4–RD, and extranodal marginal zone B–cell lymphoma of mucosa–associated lymphoid tissue (MALT lymphoma) [15,16]. A pathological analysis encompassing multiple immunohistochemical assays and cell neighbor analysis could provide insight into the immunopathogenesis of IgG4–RD developing from chronic inflammation in an extraocular muscle.

Because a subset of lymphomas develops via chronic inflammation, such as *Helicobacter pylori*–associated gastric MALT lymphoma, a subset of ocular adnexal MALT lymphomas (OAMLs) in the head–and–neck region arises from pre–existing IgG4–RD [17]. IgG4–positive OAML has clinical features similar to IgG4–RD, such as involvement of the LG, extraocular muscles, and infraorbital nerve and lymph nodes but not the conjunctiva. However, treatment outcomes are favorable despite the underlying IgG4–RD [10]. Large numbers of IgG4^+^ plasma cells are present in lymphoma, suggesting a relationship between lymphoma and IgG4–related ocular disease [15,16]. The function and mechanism of IgG4 expression in the pathogenesis of lymphoma have been investigated. However, IgG4 expression may not markedly alter the recurrence of lacrimal lymphoma. This study was limited by its retrospective design and lack of immunohistological analysis, which prevented evaluation of between–group differences according to preoperative history of glucocorticoids, ocular nerve thickening, serum IgG4 level, and prognosis [18]. Among IgG4–RD–specific causes, AID upregulation in addition to inflammation and NK–κB compounds or chromatin modifiers likely promote to develop lymphoma as drivers of oncogenesis in IgG4–RD to IgG4^+^ MALT lymphoma [18,19].

This study demonstrates that interactions between PD–L1 and immune cells in IgG4–RD are associated with treatment resistance, underscoring the importance of personalized treatment strategies. However, this study has limitations, including the sample size and the lack of coverage for various organs affected by IgG4–RD.

While PD–L1 signaling is typically immunosuppressive, its exact role in autoimmune diseases, including IgG4–RD, remains complex. Dysregulation of PD–1/PD–L1 interactions has been implicated in various autoimmune conditions, where defective PD–1 signaling leads to persistent immune activation. In IgG4–RD, the presence of PD–L1+ dendritic cells (DCs) might reflect a compensatory mechanism aimed at suppressing excessive immune activation. However, whether this contributes to disease progression or is merely a consequence of chronic inflammation remains unclear and warrants further investigation.

Moreover, we did not measure soluble PD–1 (sPD–1) or soluble PD–L1 (sPD–L1) in the current study. Given that these soluble forms can act as decoys and modulate T cell activation, future studies should investigate sPD–1/sPD–L1 in IgG4–RD to determine whether they influence disease pathogenesis or therapeutic outcomes.

Future research should include multi–center clinical trials to evaluate the efficacy and safety of PD–L1–targeted therapies, as well as explore the immunological characteristics and diagnostic criteria differences among various IgG4–RD subtypes. Additionally, studying the dynamic changes among immune cells through spatial analysis could contribute to predicting disease progression and developing new treatment strategies.

Such follow–up studies would enhance the understanding of the complex immunological features of IgG4–RD, ultimately enabling the development of personalized therapies that improve patient outcomes.

## 4. Methods and Materials

### 4.1. Tissue Material

Our study included both male and female human participants, and similar findings were observed across both sexes. IgG4 samples were obtained from patients treated at eye department in CHA university Bundang medical center. Written informed consent was obtained from all patients. The use of diseased tissues for this research was approved by the Institutional Review Board (IRB) of CHA university Bundang medical center (IRB No. 2023-04-13). Seventeen patients were diagnosed with IgG4–related ophthalmic disease including possible, probable and definite disease groups based on the 2019 ACR/EULAR Classification Criteria from April 2017 to April 2023. They were treated with steroid and other medicine such as mycophenolate mofetil, azathioprine, and hydroxychloroquine. FFPE (Formalin–Fixed Paraffin–Embedded) tissue blocks of a cohort of 17 patients were retrieved from the pathology department at CHA university Bundang medical center (including 2 normal tissues and 23 IgG4 disease tissues). Detailed clinical information and characteristics for each patient are summarized in Table 1 and Table 2. Under the supervision of H.L. and K.I.K., cores from 23 key sites of IgG4 involvement were extracted to create a tissue microarray with a core diameter of 0.3 μm. TMAs were sectioned at a thickness of 4 μm and used for tissue staining. Finally, we could successfully analyze twenty–one samples including lacrimal gland (*n* = 14), eyelids (*n* = 5) and orbit (*n* = 3), and normal samples.

### 4.2. Comparative Group Classification Criteria

In this study, the cohort of twelve patients diagnosed with IgG4–related disease in the lacrimal gland was divided into two groups based on serum diagnostic criteria and clinical symptoms including size, fibrosis, and treatment response. In categorizing patients (Ave ± SD, 362.7 ± 378.6 mg/dL) into two groups based on serum levels, tissues from 8 patients with pre–treatment serum IgG4 concentrations above 135 mg/dL were classified as high, while those from 4 patients with levels below 135 mg/dL were designated as low. For the analysis of mass size, axial length was measured from the anterior to posterior tips and axial width was measured from the widest point perpendicular to the length in orbital CT scan. The size was defined as the average of multiplications of length and width from both axial and coronal images. Twelve patients (median 35.3~278.7 mm^2^, 100.0 mm^2^) were divided into groups. The median value of 100.0 mm^2^ was used as a threshold to define the groups as either large or small. For the analysis of fibrosis, which was predominantly mild in 3 patients reviewed by K.I Kim, two criteria were established for definition. The first criterion defined regions where SMA was expressed around the Secondary Lymphoid Organ (SLO) structures as fibrotic, whereas structures showing only SLO were categorized as non–fibrotic. For treatment response, patients were defined at 6 months post–treatment. Response was defined as complete clinical and radiological resolution of ocular signs and symptoms. No response was defined as no improvement or disease worsening after 6 months of treatment. They included 8 responsive patients and 4 non–responsive patients.

### 4.3. Antibody Conjugation and Validation

We used commercial antibodies specifically intended for CODEX applications (PhenoCode Discovery Immune Profiling Human Protein Core CATALOG # PCDPC001). To ensure their performance, we validated the antibodies using immunohistochemistry (IHC) on IgG4–RD lacrimal gland tissue. This validation process focused on achieving appropriate staining intensity, clear specificity, and an acceptable signal–to–noise ratio. Antibodies that demonstrated satisfactory results under these criteria were selected. Following the manufacturer’s recommended protocols, we proceeded with the conjugation of these validated antibodies. To ensure the quality and specificity of our conjugates, we compared the staining results from the CODEX single staining on IgG4–RD in lacrimal gland tissue with those obtained using the conjugated antibodies. Additionally, a cross–validation was performed using manual IHC techniques. For further validation, we utilized the online database, The Human Protein Atlas (https://www.proteinatlas.org) (accessed on 15 October 2023). This comprehensive resource provided valuable insights, enhancing the robustness of our validation process.

### 4.4. Anti–C Reactive Protein Antibody Barcode Conjugation and Purified Antibody Reduction

Anti–C Reactive Protein [Y284]–BSA and Azide free antibody (Abcam, ab271830, Cambridge, UK) was used to create barcode conjugated antibodies for PhenoCyler–Fusion Multiplex–IHC platform (Akoya Biosciences, Marlborough, MA, USA). A total of 50 ug of CRP antibody, which is the required concentration for conjugation, was measured and calculated using IgG settings of Nanodrop (Agilent Technologies, Santa Clara, CA, USA). A 50 kDa MWCO filter was used for the purified antibody reduction process (Millipore, UFC505024, Burlington, MA, USA). Further, 500 μL of Filter Blocking Solution (Akoya Biosciences, #700009) was added to the filter and spun down at 12,000× *g* for 2 min. The remaining liquid in the filter and flow–throughs were removed. The corresponding volume for 50 ug of anti–CRP antibody was added into the MWCO filter and spun down at 12,000× *g* for 8 min to concentrate the purified antibody solution. In order to initiate the antibody reduction, Antibody Reduction Master Mix was made by mixing 6.6 μL of Reduction Solution 1 and 275 μL of Reduction Solution 2 (Akoya Biosciences, #700009). Next, 260 μL of prepared Antibody Reduction Master Mix was added to the filter unit and incubated for 25 min at RT. The tubes were spun down at 12,000× *g* for 8 min and flow–throughs were discarded. The antibody solution was exchanged by adding 450 μL of Conjugation Solution (Akoya Biosciences, #700009) to the column and having spun down at 12,000× *g* for 8 min.

### 4.5. Barcode Conjugation

CODEX Barcode–BX002 for CRP antibody was first resuspended with 10 μL of molecular biology grade water. A total of 210 μL of Conjugation Solution was added to the suspended barcode vial. The prepared CODEX Barcode Solution was transferred to the column that contained antibodies and incubated for 2 h at RT. The incubated solution was spun down at 12,000× *g* for 8 min and flow–through was discarded. Then, 450 μL of Purification Solution (Akoya Biosciences, #700009) was added into the column and spun down at 12,000× *g* for 8 min. This purification step was repeated a total of 3 times. After the purification, 100 μL of Antibody Storage Solution (Akoya Biosciences, #700009) was added into the column. While the filter contained the conjugated antibody solution, a new empty tube was placed upside down on top of the filter. Next, the filter was inverted and spun down at 3000× *g* for 2 min. Barcode conjugated antibodies were collected and stored at 4 °C for 2 days then used for staining. Conjugated antibodies were validated with gel electrophoresis following the Akoya Biosciences’ guideline prior to their usage.

### 4.6. FFPE Tissue Pre–Treatment and Antibody Staining

A IgG4–RD TMA sample slide was first baked in an incubator at 65 °C for 1 h to melt paraffin. Then, tissue deparaffinization and hydration was performed in the following order for 5 min each: Xylene, Xylene, 100% Ethanol, 100% Ethanol, 90% Ethanol, 80% Ethanol, 70% Ethanol, 50% Ethanol, 30% Ethanol, ddH_2_O, and ddH_2_O sequentially. Next, the sample slide was fixed with 4% Paraformaldehyde Solution in PBS (T&I, BPP–9004) for 1 h and washed with ddH_2_O for 5 min each twice prior to proceeding to antigen retrieval. The stock 10× AR9 Solution (Akoya Biosciences, #AR9001KT) was diluted to 1× with ddH_2_O for the antigen retrieval. Sample slide was then placed in the vessel with 250 mL of 1× AR9 buffer fully covering the slide in the pressure cooker. The cooker was set at high pressure (~11.6 PSI/110 °C) with a cooking duration of 20 min. Once the run was complete, the sample slide was taken out from the cooker and cooled down at RT for 1 h. After cooling by room temperature, the sample slide was washed with ddH_2_O and incubated for 2 min. Next, the sample slide was incubated with the Hydration Buffer (Akoya Biosciences, #7000017) for 2 min and the cycle was repeated one more time. The sample slide was then incubated with the Staining Buffer (Akoya Biosciences, #7000017) for 20 min to equilibrate the sample.

### 4.7. FFPE Tissue Staining

Antibody Cocktail Solution was prepared with 31 antibodies of interest. The following antibody panel was designed to categorize into different cell types: immune, stromal, proliferative cells, immune check–point proteins. The antibody panel consisted of the following inventoried antibodies: CD8–BX026 (Akoya Biosciences, #4250012, Marlborough, MA, USA), E–cadherin–BX014 (Akoya Biosciences, #4250021), CD14–BX037 (Akoya Biosciences, #4450047), Ki67–BX047 (Akoya Biosciences, #4250019), CD45RO–BX017 (Akoya Biosciences, #4250023), CD163–BX069 (Akoya Biosciences, #4250079), Granzyme B–BX041 (Akoya Biosciences, #4250055), CD21–BX032 (Akoya Biosciences, #4450027), CD44–BX005 (Akoya Biosciences, #4450041), CD34–BX025 (Akoya Biosciences, #4250057), Podoplanin–BX023 (Akoya Biosciences, #4250004), PD–L1 (Akoya Biosciences, #4550072), CD68 (Akoya Biosciences, #4550113), CD4 (Akoya Biosciences, #4550112), HLA–DR (Akoya Biosciences, #4550118), FOXP3 (Akoya Biosciences, #4550071), Collagen IV (Akoya Biosciences, #4550122), CD11c (Akoya Biosciences, #4550114), ICOS (Akoya Biosciences, #4550117), CD3e (Akoya Biosciences, #4550119), LAG3 (Akoya Biosciences, #4550058), CD45 (Akoya Biosciences, #4550121), PD–1 (Akoya Biosciences, #4550038), IDO1 (Akoya Biosciences, #4550123), CD20 (Akoya Biosciences, #4450018), CD31 (Akoya Biosciences, #4450017), SMA (Akoya Biosciences, #4450049), Vimentin (Akoya Biosciences, #4450050), HLA–A (Akoya Biosciences, #4450046), Pan–Cytokeratin (Akoya Biosciences, #4450020) and custom conjugated antibodies: CD57–BX029 (BioLegend, #35602, San Diego, CA, USA) (Akoya Biosciences, #5250005). The listed antibodies were kept on ice until use. Antibody Cocktail Stock Solution was brought up to a total of 300 uL by adding the following reagents in a 1.5 mL microcentrifuge tube: Staining Buffer, N–Blocker, G–Blocker, J–Blocker, and S–Blocker (Akoya Biosciences, #7000017). A total of 200 uL of Antibody Cocktail Solution was set apart, and then 57 uL of solution was removed, which is equivalent to the total antibody volume to be added. An appropriate volume of each PhenoCycler Antibody was added to the Antibody Cocktail Solution, bringing the total volume of the final solution to 200 uL (see Table 3).

The remaining 100 uL of Antibody Cocktail Stock Solution was used for antibodies with 1:500 dilution ratio. A total of 1 uL of the selected antibodies was pipetted out from each vial, then the mixture was diluted with antibody cocktail solution, bringing it up to a total of 5 uL. Next, 2 uL of diluted solution was taken and used for the final staining solution. After all of the antibodies were applied, the tube was gently vortexed. A total if 190 uL of the prepared antibody cocktail staining solution was drawn and quickly dispensed on the sample slide carefully covering the entire tissue. The sample slide was next incubated for 3 h at RT on the rocker set at 30 rpm to ensure even staining. Following the incubation step, the sample slide was additionally fixed with a 1.6% PFA Post–Staining Fixing Solution and PhenoCycler Fixative Reagent (Akoya Biosciences, #7000017), then washed with 1× PBS for use.

### 4.8. Reporter Plate Design and Preparation

Reporter Stock Solution was first prepared based on the total number of cycles for the experiment in a 15 mL amber tube. Thus, a Reporter Stock Solution required for 16 cycles was prepared with the following reagents: 1× Buffer for PhenoCycler with Buffer Additive (Akoya Biosciences, #700019), Assay Reagent (Akoya Biosciences, #7000002), and Nuclear Stain (Akoya Biosciences, #7000003), adding up to a total volume of 4.8 mL. After reagents were added, the Reporter Stock Solution was gently mixed by inverting the tube several times. Next, a Reporter Master Mix was prepared by aliquoting the Reporter Stock Solution into separate tubes following the number of reporters to be revealed for each corresponding cycle. A total of 5 uL of the Reporter Solution for each antibody was added into the corresponding cycle master mix, with a totaling volume of 250 uL. Once all of the Reporter Master Mix was prepared, 245 uL of the mixed solution for each cycle was transferred into its corresponding well in a 96–well plate (Akoya Biosciences, #7000006). Filled wells were then covered with a foil plate seal (Akoya Biosciences, #7000007).

### 4.9. Flow Cell Assembly and PCF Run

The sample slide was washed with 1× PBS and cleaned around the tissue to remove excess buffer. The flow cell was attached using the Flow Cell Assembly Device (Akoya Biosciences, #240205). The flow cell attached sample slide was then incubated in 1× PhenoCycler Buffer + Additive for 10 min. Meanwhile, PhenoCycler Designer and Fusion 2.0 corresponding to its experiment plan was set up and all the required reagents and buffer for the PhenoCycler were prepared following the given instructions from Akoya Biosciences. After the incubation, the sample slide was loaded on the PhenoCycler–Fusion to be run for Multiplex–IHC.

### 4.10. Image Preprocessing and Reprocessing

Raw images of the TMA slide were taken with PhenoCycler–Fusion. We performed preprocessing on the raw data images using Fusion software version 1.0.6. Scan Resolution was set at 0.50 μm (20×) along with the saturation protection only for the DAPI setting. Utilizing the raw and intermediate images of all 15 cycles retrieved through the PhenoImager and the Fusion software, image reprocessing was conducted by calculating the average autofluorescence signal of Atto550, Cy5, AF750 in first and last raw images (1st and 15th cycle), and then they were applied on the final multiplexed image to remove the autofluorescence. As a result, we generated a multichannel Qptiff image for a single TMA slide, devoid of autofluorescence, containing all image layers and metadata.

### 4.11. Analysis Using Oupath and Cytomap Software

QuPath software (v0.3.2)was utilized for digital pathological analysis. The Qptiff images produced from the TMA slides were analyzed following the procedure outlined in the tutorial [20]. Briefly, the analysis involved segmentation and phenotyping, followed by the classification of individual cells using the object classifier function. Subsequently, for spatial analysis, measurements such as Centroid X, Centroid Y, Image, Class, and the Cell:Mean of each marker were extracted and utilized as input data for CytoMap. In conducting detailed spatial analysis, we utilized the methods previously described by Stoltzfus et al., performing neighborhood analysis within the 2D regions/volumes of the tissue to find local configurations, thus conducting cell–cell association analysis [21].

### 4.12. Statistical Analysis

To investigate the association between diagnostic criteria and clinical symptoms in patients with IgG4–related disease, Fisher’s Exact Test was utilized. This statistical test was chosen due to the categorical nature of both the diagnostic criteria (e.g., high versus low) and the presence or absence of clinical symptoms, which are typically not amenable to analysis by parametric methods. All statistical analyses were performed using the R statistical programming language (Version 4.3.1; r–project.org).

## Figures and Tables

**Table 1:** Demography of the patients of IgG4 disease including lacrimal gland involvement.

NO.	Sex	Age	Site	Fixation	IgG4 Level (mg/dL)	Cross Sectional Area of Mass (mm^2^)	Pathologic Finding (+Fibrosis)	# of Dells with IgG4/IgG	# of IgG4 Cells/HPF	Classification	Treatment Response	Recurrence	Follow Up Duration (Months)
1	F	44	lacrimal gland	Success	91.6	162.87	absence	60	9	possible	+	-	14
2	F	74	lacrimal gland	Success	1248.0	278.67	presence	40	10	Definite	+	-	24
3	F	60	lacrimal gland	Success	93.6	35.34	presence	0	0	probable	+	-	7
4	M	71	lacrimal gland	Success	681.0	73.16	absence	40	100	Definite	+	+	51
5	M	66	lacrimal gland	Success	213.8	73.16	absence	1	1	Possible	+	-	22
6	M	60	lacrimal gland	Success	904.0	54.86	presence	40	18	Possible	+	+	44
7	M	60	lacrimal gland	Success	904.0	270.06	absence	20	48	Possible	+	-	44
8	M	19	eyelid	Success	136.6	142.33	absence	3	1	Possible	+	-	25
9	M	19	eyelid	Success	136.6	41.90	presence	0	1	Possible	+	-	25
10	F	45	lacrimal gland	Failed	93.7	69.12	absence	0	0	probable	+	-	44
11	F	45	lacrimal gland	Success	304.0	110.43	absence	90–100	10	Definite	+	+	38
12	F	45	lacrimal gland	Success	304.0	56.77	absence	90–100	10	Definite	+	-	38
13	M	76	orbit	Success	198.3	100.04	absence	0	0	Possible	+	+	38
14	F	67	eyelid	Success	99.6	386.75	absence	-	-	Possible	+	-	12
15	F	67	orbit	Success	99.6	69.12	presence	27	22	Possible	+	-	12
16	F	24	lacrimal gland	Success	171.8	268.83	absence	0	0	Possible	+	-	5
17	M	62	eyelid	Success	799.0	69.43	absence	0	0	Possible	+	-	26
18	M	62	eyelid	Success	799.0	126.10	absence	0	0	Possible	+	-	26
19	F	23	lacrimal gland	Success	110.1	56.30	absence	20	5	Possible	+	-	5
20	F	34	orbit	Failed	99.9	81.81	absence	0	0	possible	+	-	6
21	M	64	lacrimal gland	Success	136.3	6.61	absence	40	0	Definite	+	-	5
22	M	67	lacrimal gland	Success	46.6	-	absence	30	10	none	+	+	79
23	M	73	lacrimal gland	Success	99.1	188.57	presence	3	23	Definite	+	-	6

**Table 2:** IgG4–related disease: association between diagnostic criteria and clinical manifestations in lacrimal gland.

Clinicopathologic Parameter		Serum			IgG4/IgG Ratio			IgG4 10HPF		
		Low (*n* = 6)	High (*n* = 8)	*p* ^†^	40< (*n* = 7)	40≥ (*n* = 7)	*p*	10< (*n* = 6)	10≥ (*n* = 8)	*p*
Proptosis	yes	4	4	0.63	3	5	0.592	3	5	1
	no	2	4		4	2		3	3	
Size of the mass * (mm^2^)	100≥	4	3	0.59	3	5	0.592	2	6	0.28
	100<	2	5		4	2		4	2	
Fibrosis	presence	2	2	1	2	2	1	1	3	1
	absence	1	1		1	1		0	2	
Treatment response	responsive	1	3	0.58	2	2	1	0	4	0.08
	recurrent	5	5		5	5		6	4	

* Cross–sectional area of the lacrimal gland mass (mm^2^). ^†^ Fisher’s exact test (*p* < 0.05).

**Table 3:** Dilution ratio and volume requirement of antibodies for staining solution.

Antibody	Barcode	Dilution Ratio	Antibody	Barcode	Dilution Ratio	Antibody	Barcode	Dilution Ratio
CD8	BX026	1:100	PD–L1	BX043	1:200	CD20	BX007	1:200
E–cadherin	BX014	1:200	CD68	BX015	1:500	CD31	BX001	1:100
CD14	BX037	1:500	CD4	BX003	1:100	SMA	BX013	1:500
Ki67	BX047	1:500	HLA–DR	BX033	1:200	Vimentin	BX022	1:500
CD45RO	BX017	1:200	FOXP3	BX031	1:200	HLA–A	BX004	1:200
CD163	BX069	1:500	Collagen IV	BX042	1:200	PD–1	BX046	1:500
Granzyme B	BX041	1:200	CD11c	BX024	1:500	IDO1	BX027	1:500
CD21	BX032	1:500	ICOS	BX054	1:500	CD57	BX029	1:50
CD44	BX005	1:200	CD3e	BX045	1:200	PanCK	BX019	1:500
CD34	BX025	1:500	LAG3	BX055	1:500	CD45	BX021	1:500
Podoplanin	BX023	1:500						

## Data Availability

The data are not publicly available due to ethical restrictions and patient confidentiality.

## Abbreviations

IgG4–RD: IgG4–related disease; LG: lacrimal glands; IC: immune checkpoint; IHC: immunohistochemistry.
